# Initial response and 12-month outcomes after commencing dexamethasone or vascular endothelial growth factor inhibitors for retinal vein occlusion in the FRB registry

**DOI:** 10.1038/s41598-024-56581-6

**Published:** 2024-03-13

**Authors:** Gonzaga Garay-Aramburu, Adrian Hunt, Carolina Arruabarrena, Hemal Mehta, Alessandro Invernizzi, Pierre-Henry Gabrielle, Tremeur Guillaumie, Benjamin Wolff, Mark C. Gillies, Javier Zarranz-Ventura

**Affiliations:** 1 Begiker-Ophthalmology Research Group, Department of Ophthalmology, Biocruces Bizkaia Health Research Institute, OSI Bilbao Basurto, Facultad de Medicina, Campus de Basurto, University of the Basque Country, UPV/EHU, Avenida Montevideo 18, 48013 Bilbao, Spain; 2 The Save Sight Institute, Sydney Medical School, The University of Sydney, Sydney, NSW Australia; 3 Department of Ophthalmology, Westmead Hospital, Westmead, NSW Australia; 4 Department of Ophthalmology, Hospital Principe de Asturias, Madrid, Spain; 5 Ophthalmology, Royal Free London NHS Foundation Trust, London, UK; 6 Eye Clinic, Department of Biomedical and Clinical Sciences “Luigi Sacco”, University of Milan, Milan, Italy; 7 Department of Ophthalmology, Dijon University Hospital, Dijon, France; 8 Department of Ophthalmology, Saint Brieuc Hospital, 22000 Saint Brieuc, France; 9 55659 Ophthalmological Center Maison Rouge, Strasbourg, France; 10 Hospital Clinic de Barcelona, Universitat de Barcelona, Barcelona, Spain

**Keywords:** Eye diseases, Retinal diseases

## Abstract

To compare baseline characteristics, initial response and 12-month efficacy and safety outcomes in eyes with branch and central retinal vein occlusion (BRVO and CRVO) treated with dexamethasone implants (DEX) or anti-vascular endothelial growth factor (anti-VEGF) we performed a multi-centre, retrospective and observational study using Fight Retinal Blindness! Registry. Of 725 eligible eyes, 10% received DEX initially with very frequent adjunctive anti-VEGF (BRVO-DEX 49%, CRVO-DEX 60%). The primary outcome of mean adjusted change in VA at 12 months with DEX and anti-VEGF initiated groups were not statistically significantly different (BRVO: DEX + 6.7, anti-VEGF + 10.6 letters; CRVO: DEX + 2.8, anti-VEGF + 6.8 letters). DEX initiated eyes had fewer injections and visits than anti-VEGF initiated eyes. The BRVO-DEX eyes had greater initial mean changes in VA and central subfield thickness (CST) and achieved inactivity sooner than BRVO-anti-VEGF eyes. The mean CST after the first three months was above 350 μm in all but the BRVO-anti-VEGF group, suggesting undertreatment. In routine care DEX is uncommonly used when available as initial treatment of BRVO and CRVO requiring supplemental anti-VEGF within the first year. The 12-month outcomes were similar, but DEX initiated eyes had fewer injections and visits but more episodes of raised IOP Vs those starting anti-VEGF.

## Introduction

Retinal vein occlusion (RVO), the second most common retinal vascular disease^1^, may present in the form of central, hemicentral, or branch retinal vein occlusion^1–5^. The prevalence of RVO it is not influenced by gender and increases with advanced age, being estimated as 0.77% in people aged 30–89 years in 2015 data^6^. This disease is a vision-threatening disorder due not only to the presence of macular oedema (MO) but also to the development of retinal or anterior segment neovascularization.

Retinal vein occlusion generates a cascade of reactions: the capillary pressure increases, ischaemia induces the expression of vascular endothelial growth factor (VEGF) and pro-inflammatory cytokines, causing the blood-retinal barrier breakdown and the increase of vascular permeability leading MO and retinal neovascularization^7^. In the long-term, persistence and recurrence of MO leads to irreversible damage to the retina, causing low vision and/or blindness.

Therefore, anti-VEGF and anti-inflammatory drugs are the primary treatment regimens for RVO-MO. Pivotal studies^8–13^, clinical practice studies^1–25^, meta-analysis and systematic reviews^1,26–30^ have demonstrated the efficacy and safety of bevacizumab, ranibizumab, aflibercept and dexamethasone intravitreal implant (DEX; Ozurdex 0.7 mg, Allergan, an AbbVie company, North Chicago, Illinois, USA) for the MO due to RVO. However, comparative studies and meta-analyses have reported conflicting anatomical and functional outcomes^1,31–34^.

The aim of this study was to describe baseline patient characteristics and 12-month outcomes in treatment-naïve MO due to RVO initially treated with intravitreal dexamethasone implant or anti-VEGF in routine European clinical practice using the Fight Retinal Blindness (FRB)! Registry.

## Methods

### Design and setting

This retrospective observational study used anonymized data obtained from the previously described FRB! registry Retinal Vein Occlusion module^17,18^. The registry collects a prospectively defined, minimum outcome set collected via a web-based interface that does not interfere with the treatment and follow-up decisions made by treating physicians in routine care. Therefore, treatment and retreatment decisions and timing were at the discretion of the physician and patient, reflecting the clinical practice. The FRB registry has mandatory fields so, in marked contrast to other databases such as the Intelligent Research in Sight Registry (ClinicalTrials.gov Identifier: NCT02485847), anonymised data accepted by the database in the server at the University of Sydney are 100% complete and within pre-specified ranges. The study adhered to the tenets of the Declaration of Helsinki and followed the STrenghtening the Reporting of Observational studies in Epidemiology (STROBE) checklists for observational studies^35^. Ethics approval was granted in: Spain—Hospital Clínic de Barcelona (HCB/2018/0123); Italy—IRCCS Cà Granda Foundation Maggiore Policlinico Hospital; France—Société Française d’Ophtalmologie (2017_CLER-IRB_ll-05) and data protection approval in the United Kingdom—Caldicott Guardian (Until Sept 2024). Written informed consent was obtained for all patients.

### Patient selection and definitions

We included treatment-naïve patients with MO due to CRVO or BRVO that commenced treatment at FRB! centres where the dexamethasone implant (0.7 mg DEX implant; Ozurdex; Allergan, Inc, Irvine, CA) or VEGF inhibitors including ranibizumab (0.5 mg Lucentis, Genentech Inc/Novartis), bevacizumab (1.25 mg Avastin; Genentech, Inc., CA, USA/Roche, Basel, Switzerland) or aflibercept (2 mg Eylea, Bayer) were available as first-line therapy in Spain, France, Italy or the UK between March 1st, 2012, and March 1st, 2022. The study period extended from the first injection (baseline visit) until the 12-month visit (365± 30 days). Participants had at least 3 visits in the first year. Eyes with hemicentral vein occlusion were excluded. “Completers” were defined by follow-up ≥ 335 days. “Adjunctive therapy” was defined by anti-VEGF injections in DEX eyes and steroid injections in eyes initially treated with VEGF inhibitors.

### Outcomes

The primary outcome of this study was mean adjusted change in VA at first three months and at 12 months. Secondary outcomes included mean adjusted change in CST, crude changes in mean VA and CST over 12 months; visits and injections (DEX and VEGF inhibitors injections, in total and separately); non-completion; and adverse events.

### Statistical analysis

Subgroup analysis was performed by RVO type and initial treatment and included: “BRVO-DEX”, “BRVO-VEGF”, “CRVO-DEX,” and “CRVO-VEGF”. Descriptive statistics used counts, percentages, means, standard deviations (SD), medians, and first and third quartiles (Q1, Q3). Comparison of baseline demographics was conducted using Student t-test, Wilcoxon’s rank sum, and Fisher’s exact test where appropriate. We used generalised additive mixed effects models (GAMMs) to predict VA, and CST outcomes based on initial therapy with either DEX or VEGF mainly to adjust for baseline differences in eyes initially treated with DEX or VEGF. Since we observed high rates of adjunctive VEGF therapy in eyes initially treated with DEX, we took a more descriptive approach (without censorship) to present 12-month outcomes no matter what treatment was delivered. Fixed effects included age and baseline VA (or CST). Nesting of outcomes within the practice or the same patient were considered random effects. Event based outcomes were described with Kaplan–Meier survival analysis, including first grading of inactivity, first use of adjunctive therapy and non-completion. The timing of these events was compared using Cox-proportional hazards models.

Statistical analysis was performed using R version 4.1.3 (http://www.R-project.org/). Models were computed using *mgcv* (1.8–42) package. The *survival* (3.5–3) package was used to generate the Kaplan Meier estimates and *coxme* (2.2–18.1) for comparing event-based outcomes in the subgroup analysis^36^.

## Results

### Patient characteristics and disposition

We identified 725 treatment naïve eyes with BRVO (407 eyes) or CRVO (318 eyes) that started treatment at European FRB! centres where DEX and VEGF inhibitors were available as first-line therapy between March 1, 2012, and 2022. Ten percent (72/725 eyes) received the DEX implant as initial therapy, including 12% (47/407) of BRVO eyes and 8% (25/318) of CRVO eyes. The remainder were initially treated with VEGF inhibitors, including bevacizumab in 6% (41/725), ranibizumab in 50% (363/725), or aflibercept in 34% (249/725) (Table 1).

**Table 1:** Baseline characteristics of treatment naïve patients with BRVO or CRVO initially treated with DEX or VEGF inhibitors.

	BRVO-DEX	BRVO-VEGF	P value	CRVO-DEX	CRVO-VEGF	P value
Eyes, n (% of RVO type)	47 (12%)	360 (88%)		25 (8%)	293 (92%)	
Gender, % females	51%	51%	0.93	48%	48%	1.0
Age, mean years (SD)	68 (10)	70 (12)	0.40	69 (11)	72 (12)	0.22
Baseline VA, mean letters (SD)*	51 (20)	55 (21)	0.17	49 (22)	36 (27)	**0.01**
VA ≥ 70 letters, %	17%	33%	**0.04**	20%	15%	0.57
VA ≤ 35 letters, %	21%	19%	0.92	32%	47%	0.20
CST, mean microns (SD)	523 (167)	473 (157)	0.07	594 (228)	610 (226)	0.75
Hypertensive, %	49%	62%	**0.04**	72%	57%	0.25
Glaucoma, %	2%	6%	0.50	0%	13%	0.06
Pseudophakia, %	21%	17%	0.59	28%	21%	0.59
Initial agent						
Dexamethasone implant, n (%)	47 (100%)			25 (100%)		
Bevacizumab, n (%)		28 (8%)			13 (4%)	
Ranibizumab, n (%)		204 (57%)			159 (54%)	
Aflibercept, n (%)		128 (36%)			121 (41%)	

Significant values are in bold.

*Number of letters read on a logarithm of the minimum angle of resolution VA chart.

*BRVO-DEX* BRVO eyes initially treated with DEX, *BRVO-VEGF* BRVO eyes initially treated with VEGF inhibitors, *CRVO-DEX* CRVO eyes initially treated with DEX, *CRVO-VEGF* CRVO eyes initially treated with VEGF inhibitors, *VA* Visual Acuity; *CST* Central Subfield Thickness, *SD* Standard Deviation.

CRVO eyes starting DEX had better mean baseline VA than CRVO-VEGF eyes (49 versus 36 letters, respectively; *P* = 0.01). BRVO eyes starting DEX were similar in most respects to eyes starting VEGF inhibitors (Table 1), but fewer BRVO-DEX eyes had good VA ≥ 70 letters than BRVO-VEGF eyes (17% versus 33%, respectively; *P* = 0.04).

### Visual outcomes

Initial treatment response that can be attributed to DEX or VEGF inhibitors was larger in BRVO with DEX than with VEGF inhibitors at 1 and 2 months (BRVO: mean changes in VA at months 1, 2 and 3: BRVO-DEX: + 11, + 14, + 10 letters; BRVO-VEGF + 3, + 5, + 11 letters; *P* = 0.003, *P* < 0.001, *P* = 0.91, respectively). In CRVO the initial response was similar after DEX and VEGF inhibitors (Table 2) however CRVO-DEX eyes did start with significantly better baseline VA.

**Table 2:** Outcomes based on initial treatment and RVO type.

	DEX-BRVO	VEGF-BRVO	P value	DEX-CRVO	VEGF-CRVO	P value
Visual acuity (VA, letters)						
1 m Δ VA, mean (95% CI)	+ 11 (7, 16)	+ 3 (2, 4)	**0.001**	+ 6 (1, 11)	+ 3 (2, 5)	0.26
2 m Δ VA, mean (95% CI)	+ 14 (9, 19)	+ 5 (4, 6)	**0.002**	+ 5 (− 2, 13)	+ 5 (3, 7)	0.96
3 m Δ VA, mean (95% CI)	+ 10 (5, 16)	+ 11 (9, 12)	0.92	+ 2 (− 6, 10)	+ 10 (7, 12)	0.10
6 m Δ VA, mean (95% CI)	+ 9 (4, 13)	+ 11 (10, 13)	0.28	+ 3 (− 7, 13)	+ 8 (5, 11)	0.37
12 m Δ VA, mean (95% CI)	+ 6 (0, 12)	+ 11 (9, 13)	0.18	+ 0 (− 10, 10)	+ 7 (5, 10)	0.16
12 m adjusted Δ VA, mean (95% CI) *	+ 6.7 (+ 1.8, + 11.7)	+ 11.7 (+ 9.2, + 13.2)		+ 2.8 (− 6.8, + 12.4)	+ 6.8 (+ 3.3, + 10.3)	
12 m gained ≥ 15 letters, %	34%	36%	0.93	20%	35%	0.18
12 m lost ≥ 15 letters, %	15%	6%	0.07	20%	15%	0.56
Central subfield thickness (CST, μm)						
1 m Δ CST, mean (95% CI)	− 162 (− 210, − 113)	− 46 (− 59, − 33)	** < 0.001**	− 144 (− 247, − 42)	− 65 (− 85, − 44)	0.15
2 m Δ CST, mean (95% CI)	− 215 (− 269, − 160)	− 61 (− 75, − 48)	** < 0.001**	− 231 (− 333, − 130)	− 99 (− 123, − 76)	**0.02**
3 m Δ CST, mean (95% CI)	− 135 (− 193, − 77)	− 135 (− 153, − 117)	0.99	− 195 (− 288, − 102)	− 188 (− 218, − 159)	0.89
6 m Δ CST, mean (95% CI)	384 (159)	328 (109)	0.71	390 (155)	387 (216)	0.97
12 m Δ CST, mean (95% CI)	− 110 (− 167, − 53)	− 143 (− 161, − 126)	0.28	− 217 (− 317, − 117)	− 192 (− 221, − 164)	0.65
12 m adjusted Δ CST, mean (95% CI) *	− 107 (− 151, − 62)	− 155 (− 172, − 138)		− 207 (− 276, − 139)	− 248 (− 280, − 215)	
12 m completers, n (%)	40/47 (85%)	288/360 (80%)		22/25 (88%)	226/293 (77%)	0.19
Injections and visits						
Total injections, median (Q1, Q3)**	4 (3, 5)	7 (5, 8)	** < 0.001**	3 (3, 6)	7 (5, 9)	** < 0.001**
DEX injections, mean	2.1	0.1		1.8	0.2	
VEGF injections, mean	1.8	5.9		2.2	6.2	
Visits, median (Q1, Q3)**	9 (7, 11)	10 (8, 12)	0.15	9 (7, 11)	12 (9, 14)	**0.002**
Adjunctive therapy used, n (%)^#^	23 (49%)	37 (10%)	** < 0.001**	15 (60%)	31 (11%)	**0.002**
VEGF injections, median (Q1, Q3)**	1 (0, 3)	6 (4, 8)		2 (0, 4)	7 (4, 9)	
DEX injections, median (Q1, Q3)**	2 (1, 3)	0 (0, 0)		2 (1, 2)	0 (0, 0)	
Additional treatment & adverse outcomes						
Sectoral or PRP laser, n (%)	5 (11%)	52 (14%)	0.65	3 (12%)	129 (44%)	0.03
Focal/macular laser, n (%)	1 (2%)	6 (2%)	0.58	0 (0%)	3 (1%)	1.0
Elevated IOP req. treatment, n (%)	5 (11%)	9 (2%)	**0.015**	4 (16%)	17 (6%)	0.07
Cataract surgery, n (%)	2 (4%)	23 (6%)	0.75	1 (4%)	16 (5%)	1.0
Neovascular complications, n (%)***	1 (2%)	7 (2%)	1.0	2 (8%)	31 (11%)	0.94
Neovascular glaucoma specifically, n (%)	0 (0%)	1 (0%)	1.00	1 (4%)	22 (8%)	0.80
Macular changes affecting vision, n (%)****	7 (15%)	47 (13%)	0.90	4 (16%)	39 (13%)	0.94

Significant values are in bold.

*Generalised mixed effects models were used for adjustment and 95% confidence intervals: Fixed effects—baseline VA (CST) and age. Random effects—nesting in practices and bilateral disease. The confidence intervals all overlapped at 12 months despite significant differences at other times in the models in both BRVO (VA: *P* < 0.001, CST: *P* < 0.001) and CRVO (VA: *P* = 0.047, CST: *P* < 0.001) eyes initially treated with DEX or VEGF.

**Total injections of any drug calculated on Completers only ***Neovascular Complications in either the anterior or posterior segment ****Macular hole/Epiretinal membrane/Pigmentary macular changes judged by the treating physician as affecting visual acuity.

*BRVO-DEX* BRVO eyes initially treated with DEX, *BRVO-VEGF* BRVO eyes initially treated with VEGF inhibitors, *CRVO-DEX* CRVO eyes initially treated with DEX, *CRVO-VEGF* CRVO eyes initially treated with VEGF inhibitors, *Δ* Change; *1 m, 2 m, 3 m, 12 m* 1-, 2-, 3-, 12-month, *n* number, *VA* Visual Acuity in letters read on a logarithm of the minimum angle of resolution VA chart (best of uncorrected, corrected or pinhole), *SD* Standard Deviation, *CI* Confidence interval, *Q1, Q3* Interquartile range, *PRP* Panretinal photocoagulation (number of patients that received it rather than the number of treatments), *IOP* Intraocular pressure where req. treatment included selective laser trabeculoplasty or topical antihypertensive agents.

**Figure 1:**
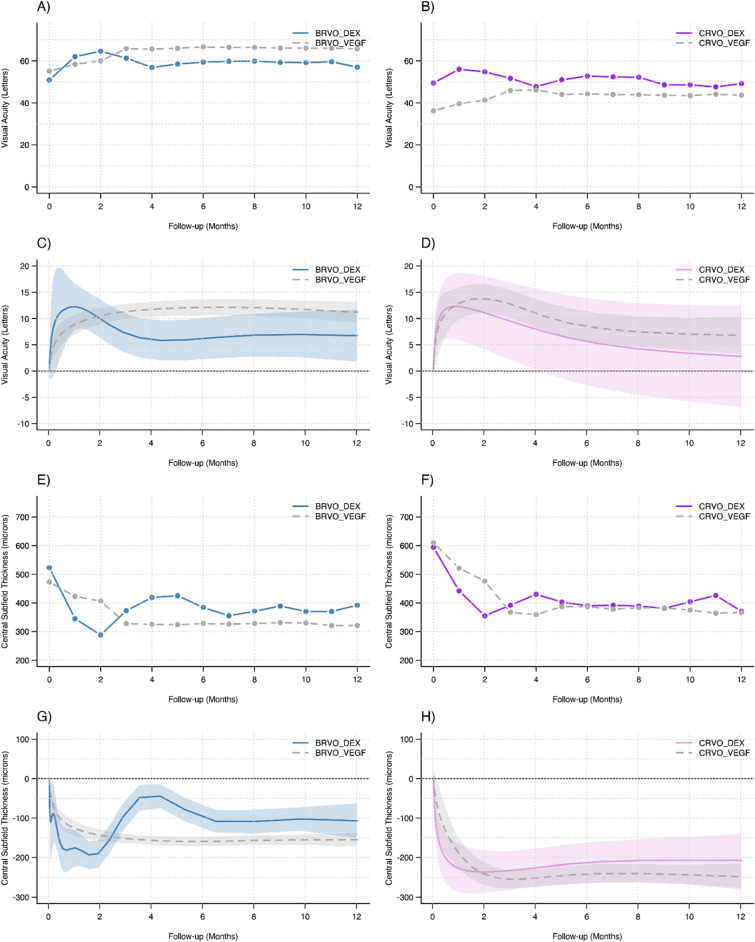
Unadjusted and Adjusted outcomes by RVO type after initial treatment with DEX or VEGF inhibitors. Unadjusted plots include mean VA (**A**, **B**) and mean CST (**E**, **F**). Generalised additive mixed effects models were generated to plot Adjusted Change in VA (**C**, **D**) and Adjusted Change in CST (**G**, **H**) with 95% confidence intervals shaded. The confidence intervals all overlapped at 12 months despite significant differences at other times.

12-month outcomes reflect combination therapy in many eyes, particularly the DEX groups because of widespread adjunctive VEGF inhibitors therapy during the 12-month study. Nevertheless, the primary outcome of mean 12-month adjusted change in VA (95% CI) in BRVO eyes was + 6.7 (+ 1.8, + 11.7) letters when initially treated with DEX and + 11.7 (+ 9.2, + 13.2) letters with VEGF inhibitors; in CRVO it was + 2.8 (− 6.8, + 12.4) letters when initially treated with DEX and + 6.8 (+ 3.3, + 10.3) letters with VEGF inhibitors. The confidence intervals overlap at 12 months in Fig. 1C and D.

### Anatomical outcomes

Initial anatomical response was larger in BRVO with DEX than with VEGF inhibitors at 1 and 2 months (DEX-BRVO: mean changes in CST at months 1, 2 and 3: DEX: − 162 μm, − 215 μm, − 135 μm; VEGF-BRVO: − 46 μm, − 61 μm, − 133 μm*; P* < 0.001, *P* < 0.001, *P* = 0.99, respectively). The trend reversed significantly in favour of VEGF inhibitor-initiated eyes between 3 and 10 months (Fig. 1G). Despite BRVO-DEX eyes having the lowest mean CST of any group during the study, 282 μm at 2 months, mean CST was 395 μm at 4 months and stayed at or above 350 μm for the remainder of the year. The macular thickness in BRVO-VEGF was better maintained at around 325 μm from 3 to 12 months (Fig. 1E).

The DEX and VEGF inhibitors initiated CRVO eyes had similar early unadjusted reductions in CST with the exception of greater change in CST with DEX at month 2 (DEX-CRVO: mean changes in CST at months 1, 2, and 3: DEX: − 144 μm, − 231 μm, − 195 μm; VEGF-CRVO: − 65 μm, − 99 μm, − 188 μm*; P* = 0.15, *P* = 0.02,* P* = 0.89, respectively). After 3 months, the mean CST was generally higher in CRVO-DEX eyes than in CRVO-VEGF eyes (Fig. 1). In both CRVO groups, the mean CST generally remained above 350 μm during the remainder of study (Fig. 1C,D).

The 12-month adjusted mean change in CST (95% CI) in BRVO-DEX eyes was − 107 μm (− 151, − 62) and in BRVO-VEGF eyes was − 155 μm (− 172, − 138), in CRVO-DEX it was − 207 μm (− 276, − 139), and in CRVO-VEGF eyes it was − 248 μm (− 280, − 215) with overlapping confidence intervals in both BRVO and CRVO at 12 months (Fig. 1G,H).

### Injections, visits

The BRVO-DEX 12-month completers (40/47 [85%]) had a median (Q1, Q3) of 4 injections (3, 5) in total, fewer than the BRVO-VEGF eyes with 7 injections (5, 8) (*P* < 0.001) but we found no significant difference in the frequency of visits in BRVO groups (median, DEX, 9 visits; VEGF, 10 visits; *P* = 0.15). The CRVO-DEX 12-month completers (22/25 [88%]) had a median (Q1, Q3) of 3 injections (3, 6) in total and 9 visits (7, 11), fewer than the 7 injections (5, 9) and fewer than the 12 visits (9, 14) in CRVO-VEGF eyes (*P* < 0.001,* P* = 0.002). The median (Q1, Q3) time between first and second DEX injections was 175 days (129, 243).

### Inactivity, adjunctive therapy, and non-completion

**Figure 2:**
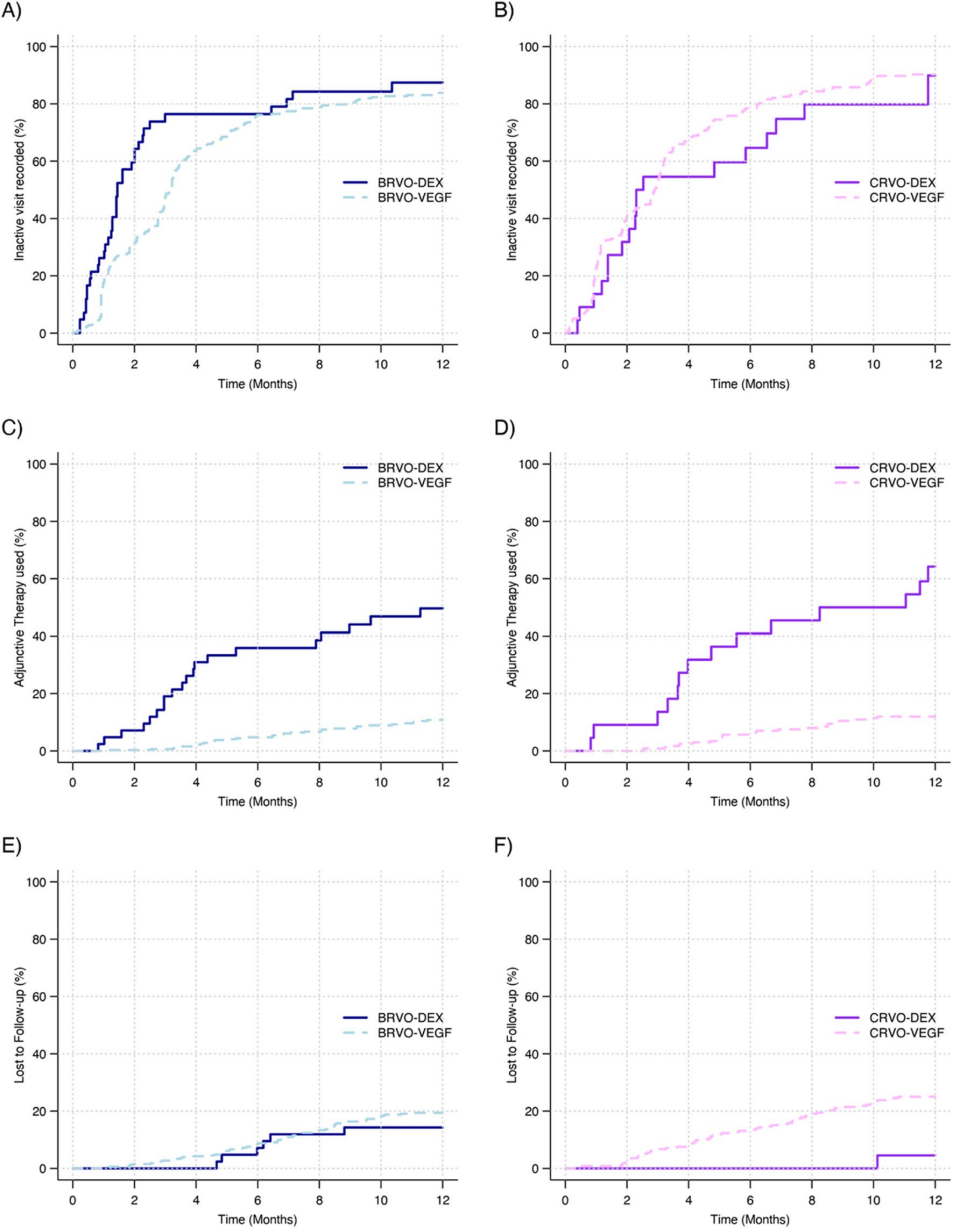
Kaplan Meier survival curves for time to first grading of inactivity, use of adjunctive therapy (VEGF in DEX eyes/DEX in VEGF eyes), and loss to follow-up by RVO type and initial treatment with DEX or VEGF inhibitors.

Time to first grading of inactivity, adjunctive therapy and loss to follow-up were analysed with Kaplan–Meier estimates by initial treatment with DEX or VEGF inhibitors (Fig. 2). BRVO-DEX eyes were more likely to achieve inactivity than BRVO-VEGF eyes after adjustment with Cox-proportional hazards models (*P* = 0.01), no significant difference was found in CRVO (*P* = 0.9). The proportion of DEX initiated eyes receiving adjunctive VEGF inhibitors therapy with BRVO was 49% and with CRVO 60% compared with adjunctive DEX therapy in BRVO-VEGF and CRVO-VEGF eyes (both 10%; both *P* < 0.001, Fig. 2). Table 3 describes outcomes in DEX initiated eyes that appeared to benefit from additional VEGF inhibitors rescue therapy compared with those remaining on DEX monotherapy. Non-completion rates were similar (Fig. 2, BRVO-DEX 7/47 [15%], BRVO-VEGF eyes 72/360 [20%]; *P* = 0.17, and CRVO-DEX 3/25 [12%], CRVO-VEGF 67/293 [23%]; *P* = 0.19).

**Table 3:** Outcomes in BRVO-DEX and CRVO-DEX eyes based on treatment received being DEX monotherapy or a combination of DEX and Adjunctive VEGF therapy.

	BRVO-DEX DEX monotherapy	BRVO-DEX adjunctive VEGF therapy	CRVO-DEX DEX monotherapy	CRVO-DEX adjunctive VEGF therapy
12 m completers/eyes, n (%)	20/24 (83%)	20/23 (87%)	9/10 (90%)	13/15 (87%)
Visual acuity (VA, letters)				
1 m Δ VA, mean (95% CI)	+ 9 (4, 14)	+ 13 (5, 21)	+ 5 (− 3, 13)	+ 7 (1, 14)
2 m Δ VA, mean (95% CI)	+ 13 (8, 18)	+ 15 (5, 24)	+ 2 (− 9, 12)	+ 8 (− 3, 18)
3 m Δ VA, mean (95% CI)	+ 8 (3, 14)	+ 12 (4, 21)	+ 1 (− 11, 13)	+ 3 (− 8, 14)
6 m Δ VA, mean (95% CI)	+ 4 (− 2, 11)	+ 13 (6, 20)	+ 7 (− 9, 22)	− 2 (− 11, 7)
12 m Δ VA, mean (95% CI)	+ 2 (− 5, 9)	+ 10 (0, 21)	− 5 (− 18, 8)	+ 3 (− 11, 17)
12 m gained ≥ 15 letters, %	21%	48%	10%	27%
12 m lost ≥ 15 letters, %	17%	13%	30%	13%
Central subfield thickness (CST, μm)				
1 m Δ CST, mean (95% CI)	− 168 (− 241, − 95)	− 155 (− 220, − 90)	− 157 (− 280, − 34)	− 136 (− 289, 17)
2 m Δ CST, mean (95% CI)	− 210 (− 282, − 138)	− 220 (− 303, − 137)	− 231 (− 339, − 123)	− 231 (− 388, − 75)
3 m Δ CST, mean (95% CI)	− 135 (− 217, − 53)	− 135 (− 217, − 53)	− 224 (− 327, − 121)	− 176 (− 317, − 34)
6 m Δ CST, mean (95% CI)	− 124 (− 195, − 53)	− 142 (− 239, − 44)	− 265 (− 433, − 96)	− 159 (− 241, − 77)
12 m Δ CST, mean (95% CI)	− 121 (− 196, − 46)	− 98 (− 185, − 12)	− 113 (− 189, − 36)	− 286 (− 437, − 135)
Injections and visits				
Total injections, median (Q1, Q3)**	2 (2, 3)	6 (4, 6)	2 (2, 3)	5 (4, 6)
DEX injections, mean	2.3	1.8	2.3	1.5
VEGF injections, mean	0	3.7	0	3.7
Visits, median (Q1, Q3)**	8 (7, 11)	10 (8, 11)	8 (7, 9)	9 (7, 12)
Adjunctive therapy used, n (%)^#^	0 (0%)	23 (100%)	0 (0%)	15 (100%)
VEGF injections, median (Q1, Q3) **	0 (0, 0)	3 (2, 5)	0 (0, 0)	4 (2, 5)
DEX injections, median (Q1, Q3) **	2 (2, 3)	2 (1, 3)	2 (2, 3)	1 (1, 2)
Additional treatment & adverse outcomes				
Sectoral or PRP laser, n (%)	3 (12%)	2 (9%)	1 (10%)	2 (13%)
Focal/macular laser, n (%)	1 (4%)	0 (0%)	0 (0%)	0 (0%)
Elevated IOP req. treatment, n (%)	2 (8%)	3 (13%)	0 (0%)	4 (27%)
Cataract surgery, n (%)	2 (8%)	0 (0%)	1 (10%)	0 (0%)
Neovascular complications, n (%)***	0 (0%)	1 (4%)	0 (0%)	2 (13%)
Neovascular glaucoma specifically, n (%)	0 (0%)	0 (0%)	1 (7%)	0 (0%)
Macular changes affecting vision, n (%)****	3 (12%)	4 (17%)	3 (30%)	1 (7%)

**Total injections of any drug calculated on Completers only ***Neovascular Complications in either the anterior or posterior segment ****Macular hole/Epiretinal membrane/Pigmentary macular changes judged by the treating physician as affecting visual acuity.

*Δ* Change, *1 m, 2 m, 3 m, 12 m* 1-, 2-, 3-, 12-month, *n* number, *VA* Visual Acuity in letters read on a logarithm of the minimum angle of resolution VA chart (best of uncorrected, corrected or pinhole), *SD* Standard Deviation, *CI* Confidence interval, *Q1, Q3* Interquartile range, *PRP* Panretinal photocoagulation (number of patients that received it rather than the number of treatments), *IOP* Intraocular pressure where req. treatment included selective laser trabeculoplasty or topical antihypertensive agents.

### Adverse outcomes

We found a higher rate of elevated IOP requiring treatment in BRVO (DEX 5/47 [11%] vs. VEGF inhibitors treated eyes 9/360 [2%]; *P* = 0.015). Elevations in IOP of > 10 mmHg from baseline IOP occurred more frequently in DEX eyes (42/80 [52%], at a median [Q1, Q3] 109 days [70, 199]) compared with VEGF eyes (79/721 eyes [11%]; P < 0.01, at a median [Q1, Q3] of 168 days [112, 253]). The earliest occurrence of an elevation in IOP of > 10 mmHg occurred 25 days after a DEX injection. No other significant difference in cataract surgery, new macular changes affecting vision (epiretinal membrane, macular hole, pigment clumping, or atrophy) or neovascular complications based on initial DEX or VEGF inhibitors treatment in BRVO or CRVO. The higher rate of PRP in CRVO-VEGF eyes reflected individual practice patterns from one large practice centre that mainly used VEGF inhibitors as initial therapy. Overall, a total of 4311 injections (including 239 DEX-implants) were delivered in the study with one retinal detachment (BRVO-VEGF), one iatrogenic cataract (BRVO-VEGF), and one case of infectious endophthalmitis (CRVO-VEGF).

## Discussion

This observational study using data from the prospectively designed RVO module of the FRB! registry recruited eyes at European centres where both VEGF inhibitors and DEX were available for treatment naïve MO due to RVO found that DEX was used in only around 10% of eyes but outcomes at 12 months were similar to the majority that were initially treated with VEGF inhibitors. Our analysis adjusted for baseline differences including significantly higher VA in CRVO eyes initially treated with DEX rather than VEGF inhibitors. We found no significant difference in the primary outcome at 12 months of mean adjusted change in VA based on initial treatment with DEX or VEGF inhibitors of BRVO (DEX + 6.7 letters, BRVO + 11.7 letters) or CRVO (DEX + 2.8 letters, VEGF + 6.8 letters). Significant secondary outcomes included higher rates of adjunctive therapy use after initial DEX (BRVO 49% and CRVO 60% VEGF inhibitors use) than initial VEGF inhibitors (10% DEX use), inactivity earlier in BRVO after DEX than VEGF inhibitors; fewer injections in both BRVO and CRVO after DEX than VEGF inhibitors; fewer visits in CRVO after DEX than VEGF inhibitors; and higher rate of elevated IOP requiring treatment in BRVO after DEX than VEGF inhibitors.

We found no significant difference based on initial treatment with DEX or VEGF inhibitors in 12-month mean adjusted change in CST in BRVO or CRVO, time to inactivity in CRVO, frequency of visits in BRVO. About three quarters of eyes were phakic at baseline and there was no significant difference in rates of cataract surgery in the first year of treatment, although other comparative studies of retinal vascular disease have identified higher rates of cataract surgery with DEX from the second year^22^.

The 12-month outcomes in this study were not a comparison of VEGF inhibitors and DEX monotherapy as there was widespread adjunctive VEGF inhibitors after initial treatment with DEX. This led us to analyse initial response that could be attributed uniquely to DEX or VEGF inhibitors. In CRVO, even though DEX initiated eyes had higher baseline VA, the mean change in VA at 2 months was larger than CRVO-VEGF eyes. In BRVO we observed impressive initial response to DEX with significantly larger improvements in mean VA and CST at months 1 and 2, and inactivity was achieved earlier with DEX than with initial VEGF inhibitors.

The efficacy of initial DEX waned beyond 3 months in both BRVO-DEX and CRVO-DEX groups, likely compromising 12-month outcomes. BRVO-DEX eyes were most affected with mean CST almost returning to baseline by 4 months with limited recovery subsequently. The CRVO-DEX group had a similar set back in mean CST at 4 months, but a higher proportion received rescue VEGF inhibitors injections. The VEGF inhibitors initiated groups achieved improvements more gradually and better maintained them through 12 months.

This study adds real-world evidence to pivotal trial evidence suggesting DEX therapy usually needs be delivered more frequently than 6-monthly in RVO to maintain outcomes. The saw-tooth pattern in mean CST in the GENEVA study suggested efficacy of DEX waned before 6 months. The proportion of patients with VA ≥ 15 letters from baseline was maximal at day 30 (15.0%) and day 60 (17.6%) but the study failed to demonstrate a difference between DEX and sham at 180 days (6.5% [95% CI − 0.9% to 13.9%])^8^. Nevertheless, this led to approval of DEX as initial therapy in RVO in Italy, France, Spain, and the UK with an initial 6-month limit on re-treatment. It remains unclear if more frequent retreatment using DEX monotherapy could make it a more viable initial treatment option in RVO. We believe the widespread uptake of adjunctive VEGF inhibitors therapy after initial DEX treatment reflects an attempt to counter undertreatment associated with what was typically a 6-month treatment interval between injections of DEX in this study.

Generalised undertreatment in this study also likely affected outcomes in all but the BRVO-VEGF group. The BRVO-DEX, CRVO-DEX and CRVO-VEGF groups consistently had mean CST above 350 μm in the latter part of the study. Only the BRVO-VEGF group maintained mean CST around 325 μm from 3 to 12 months. Less impressive outcomes in routine care are often blamed on treatment burden. Even though fewer injections and visits were observed after starting DEX, it was subsequent undertreatment that likely explains the less impressive 12-month VA and CST outcomes in this study compared with pivotal studies^8–11,13^ and other real-world studies^19,20,31–34^. The baseline VA was generally better in this study than previous reports from our group^17,18^. In particular, the high baseline VA in the CRVO-DEX eyes would have left less room for improvement, though we did adjust for that.

Ocular hypertension is a known side effect of intravitreal dexamethasone implant occurring at 6 to 8 weeks and returning to baseline by around 3 to 4 months and could be managed by topical anti-glaucoma medications in 90% eyes (14,26). Similar too previous studies (14, 26) we found a higher rate of elevated IOP requiring treatment in the DEX initiated eyes compared with VEGF initiated eyes (BRVO-DEX 5/47 [11%] vs. BRVO-VEGF 9/360 [2%]; *P* = 0.015; and CRVO-DEX 4/25 [16%] vs CRVO-VEGF 17/293 [6%]; P = 0.07). The data field “IOP elevations requiring treatment” collected in the registry encompasses a wider variety of definitions (i.e. IOP ≥ 25/30/35, IOP change ≥ 5/10/15, etc.) and in our opinion is more clinically relevant, as it takes into account individual considerations in each specific case (i.e. glaucoma eyes, cupped discs, etc.), although we do acknowledge that this may contribute to explain slight differences to benchmark our data with other series. Mun et al.^23^ considered that the side effects of steroids caused physicians consider anti-VEGF agents as a first-line drug and we think that could be a cause of delay in treatment or even undertreatment as physician dedicate follow-up visits to treating adverse effects and leave aside the treatment of macular edema until the adverse effect resolved.

In the absence of any other difference, such as macular changes affecting vision, we do believe that the numerically higher rate of losing ≥ 15 letters in the DEX groups was likely due to development of cataract secondary to the use of intraocular steroid. It is not surprising though that the rates of cataract surgery were similar in DEX and VEGF groups because the study spanned only first 12 months after therapy commenced leaving little time to schedule and complete cataract surgery. Longer term follow-up with more patients would be required to confirm this point as Garay-Aramburu et al.^22^ reported in a five-year follow-up study. Furthermore, we observed no statistically significant differences between both groups regarding additional treatments and adverse outcomes, which included focal and PRP laser, cataract surgeries, neovascular complications, neovascular glaucoma and macular changes affecting vision. We do believe that the limited 12 months timespan of the study could also explain these results.

This study has inherent weaknesses associated with the use of observational data from a real-world database^16–20,27^. In contrast to randomized controlled trials (RCT), treatment and retreatment decisions including timing are based on the physician´s observation and in accordance with the patient, resulting in a heterogeneity of treatment. This heterogeneity is influenced by the introduction of drugs, their efficacies and cost, the resources at the different sites, burden related to treatment^23,29^, the span of the study and the differences between Clinical Guidelines^2,4^; but, despite this heterogeneity, the outcomes reported by real-world studies contribute to these Guidelines^23^.

As with other real-world studies^16–20,27^, several RCTs^1,31,32^ and meta-analyses^28–30^, our dataset did not differentiate between ischemic and non-ischemic RVO when reporting outcomes. Ang et al.^27^ reviewed 48 real-world studies of BRVO. Because of generally poor reporting of ischaemia in the 71 treatment arms included in that meta-analysis, whether macular or peripheral, they found divergent results regarding the effect of ischaemia on visual acuity gains at least in BRVO when treated with intravitreal therapy. Our study reported that 44% of the CRVO-VEGF patients received PRP—a relatively high proportion—manly because one centre seemingly performing it routinely. There is no consensus on routine delivery of PRP in CRVO within the clinical guidelines^2,4^. Li et al.^37^ published a systematic review indicating that laser photocoagulation did not appear to be effective in modifying the visual acuity outcomes. The implication being that PRP should probably be reserved for neovascular complications. Nevertheless, that study suggests that the visual outcomes were likely not influenced by having one centre perform PRP at a higher rate than usual. The weaknesses of the retrospective nature of our study were compensated for to some extent by the strength of the FRB registry that forces the completion of all fields within pre-specified ranges ensuring data integrity. Besides, follow-up was excellent compared with similar studies^33^, but we were unable to fairly compare DEX and VEGF inhibitors over 12-months because of the confounding effect of adjunctive VEGF inhibitors therapy.

The strength of this study is the comparison of initial response to DEX compared with VEGF. The study also identifies how adjunctive VEGF inhibition is required in order to salvage outcomes when a limit of 6 months is placed on DEX retreatment. More frequent retreatment would be required for DEX monotherapy to become a viable alternative to VEGF inhibitors in RVO while still possibly reduce burden of therapy, although the impact on rates of raised intraocular pressure would need to be considered.

In conclusion, this study identified infrequent use of DEX as initial therapy in routine care for RVO despite superior initial response particularly in BRVO compared with VEGF inhibitors. Outcomes were similar at 12 months in both BRVO and CRVO after DEX or VEGF inhibitors as initial treatment. Following initial treatment with DEX, subsequent outcomes suffered due to the 6-month limit on DEX retreatment necessitating frequent VEGF inhibitors rescue therapy. The real-world practice patterns detailed in this manuscript suggest that more robust treatment strategies are required to optimize the clinical outcomes in RVO patients.

## Data Availability

The dataset used and analyzed during the current study are available from the corresponding author on reasonable request.
